# A CT-Scan review of anatomical variants of sinonasal region and its correlation with symptoms of sinusitis (nasal obstruction, facial pain and rhinorrhea)

**DOI:** 10.12669/pjms.37.1.3260

**Published:** 2021

**Authors:** Maryam Faiz Qureshi, Ambreen Usmani

**Affiliations:** 1 Dr. Maryam Faiz Qureshi, MBBS, Postgraduate M.Phil. student. Department of Anatomy, Bahria University Medical and Dental College, Karachi, Pakistan; 2 Prof. Dr. Ambreen Usmani, Ph.D. (Anatomy), HOD, Principal, Department of Anatomy, Bahria University Medical and Dental College, Karachi, Pakistan

**Keywords:** Anatomical variations, CT-scan, Facial pain, Nasal obstruction, Sinonasal region, Sinusitis, Rhinorrhea

## Abstract

**Objective:**

To determine the incidence of anatomical variants of sinonasal region and its correlation with symptoms of sinusitis.

**Methods:**

The study was conducted from January-June 2020 at Radiology Department of PNS Shifa Hospital, Karachi. The study involved 50 symptomatic subjects of sinusitis with age ranges from 18-60 years which were prepared for computed tomography of paranasal sinuses. The scans were reviewed for nasal-septum, turbinates, uncinate process, ethmoid air-cells along with other anatomical variants and were correlated with symptoms of sinusitis.

**Results:**

Out of 50 subjects, 34 were males and 16 were females with mean age of 42.68±18.22 years. Most common anatomical variants observed were agger nasi cells (64%), deviated nasal septum (56%), and concha-bullosa (46%). Statistically significant correlation existed between bilateral agger nasi cells and nasal obstruction (p=0.017, ρ= -0.336).

**Conclusion:**

The anatomy of sinonasal region is highly complex. However, anatomical variants can disturb the sinus mucociliary drainage pathway resulting in patient suffering. Therefore, considering the variable anatomy of sinonasal region, CT-PNS is recommended for every subject in order to avoid surgical hazards.

## INTRODUCTION

The paranasal sinuses (PNS) are basically air-filled cavities present in facial and skull bones. Sinuses are named for the bones in which they are located: maxillary sinus, sphenoid sinus, ethmoid sinus, and frontal sinus. The PNS reduce the weight of the head, cause air humidification, and aid in voice resonance.^1^

There are many anatomic variants in sinonasal region which are frequently observed on computed tomography (CT). Out of which most frequently observed anatomical variants are agger nasi cells, deviated nasal septum (DNS), infraorbital ethmoidal (Haller) cells, sphenoethmoidal (Onodi) cells, and concha-bullosa (CB). The most anterior ethmoidal air-cells are agger nasi cells which are located anterior and inferolateral to frontal recess. The infraorbital ethmoidal cells extend downward underneath the medial floor of orbit lying adjacent to and above the ostium of maxillary sinus and lateral to the infundibulum. The posterior ethmoidal-cells extend superolateral, and posterior to the sphenoid sinus cavity, these cells are closely associated with CN-II. DNS is defined as any bending of the nasal-septa. CB is defined as pneumatization (formation of air-cells) of middle turbinate, mostly occur bilateral and inferior bulbous part is involved. It has been reported that failure to spot various anatomic variations can lead to complications during surgery.^2^

Some organs of human-body are subject to notable anatomical variations as in nasal cavity and PNS. The vast range of anatomic variants can hinder with mucociliary drainage pathway of osteomeatal complex (OMC) which includes DNS, CB, paradoxical middle turbinate, uncinate process (UP) variants, ethmoid bulla, various ethmoidal air-cells and many others. Enhancements in functional endoscopic sinus surgery (FESS) and radiographic CT-scans have concomitantly amplified interest in variation of nasal cavity and PNS region anatomy.^3^ OMC is the crucial area for spread and pathogenesis of rhinosinusitis. Episodes of rhinosinusitis hinders the movement of cilia that results in collection of mucous within sinuses. However, if there is an anatomical variant, causing narrowing of key area which is OMC, then a minimal amount of mucosal distention may predispose to recurrent infections and may result in severe inflammatory changes in the mucus membrane of sinonasal region.^4^,^5^

Although the role of anatomical variants of OMC in etiology of sinonasal pathologies is controversial but the detailed knowledge of anatomical variants in every subject is vital before planning for surgery in order to avoid injury to surrounding vital structures such as orbit and brain. CT-PNS and FESS have these days become choice of management modalities for radiological diagnosis and treatment of sinonasal morphology along with pathologies.^6^-^8^

In the literature search, no prospective data was reported on anatomical variations of nasal cavity and PNS, in patients having symptoms of sinusitis (nasal obstruction, facial pain, and rhinorrhea) in local population of Karachi. This study was done to report the frequency of anatomical variations of sinonasal region in symptomatic patients who underwent CT-PNS in PNS Shifa Hospital, Karachi. The objective of this study was to:

1. Detect and identify anatomical variations of sinonasal region on CT-scan.
2. Determine the correlation of anatomic variants with symptoms of sinusitis including nasal obstruction, facial pain and rhinorrhea.

## METHODS

A cross-sectional study was conducted at Radiology Department of PNS Shifa Hospital, Karachi between January-June 2020. Before commencing the research, ethical approval (Ref. No. 05/2020 January 9^th^ 2020) was obtained from the Ethical Review Committee of Bahria University Medical and Dental College, Karachi. The sampling technique was Non-Probability convenience sampling. The sample size was calculated by open-epi version-3 calculator and it was found to be 50. The participants were symptomatic subjects of sinusitis irrespective of gender with complains of nasal obstruction, facial pain and rhinorrhea, which were referred from ENT Department of PNS Shifa hospital, Karachi were included in the study.

### Inclusion criteria:

1. Symptomatic subjects of sinusitis (nasal obstruction, facial pain, and rhinorrhea)
2. Age ranges from 18-60 years
3. Radiological features: mucosal thickening of greater than 1 mm.

### Exclusion criteria:

1. Subjects below 18 years
2. Mucosal thickening of less than one mm
3. Fungal-sinusitis
4. Sinonasal polyposis
5. Sinonasal malignancy
6. Pregnant females
7. Previous sinus surgery
8. Facial-Trauma

### CT-scan protocol

Subjects meeting the inclusion criteria were considered after written informed consent. Detailed history regarding age, symptoms was taken from all subjects and were prepared for CT-PNS in the coronal plane, supplemented by axial views. CT-scan of Prime Aquilion-160 slice of Toshiba company was used. For coronal view, images were taken in prone position. Hard-palate was the reference point; plane of section was perpendicular to it. Direct scans of 3-mm in thickness were made, from the anterior walls of the frontal sinuses to the posterior wall of the sphenoid sinus. For axial-scans, slice thickness was 3-mm thick, the orbitomeatal line was the reference point in supine position. The exposure settings used as 120 kV and 80 mAs. After CT reporting of subjects, all anatomical variations of sinonasal region were noted.

In all cases, following anatomical variants were studied:

- ***Nasal-septum:*** septal deviation, bony spur
- ***Turbinate:*** superior CB, middle CB, paradoxical middle concha, hypoplasia, and secondary middle concha
- ***Uncinate-process:*** deviation of upper edge, pneumatization
- ***Ethmoid air-cells:*** agger nasi, haller, onodi
- ***Other variants:*** hypoplasia of maxillary sinus, maxillary-septa, hypoplastic frontal sinus and asymmetry of both cavities of sphenoid sinus.

### Statistical Analysis

For quantitative variables like age mean and standard deviation was determined. For qualitative variables like gender, symptoms and anatomical variants frequencies with percentages were computed. SPSS version 23.0 was used for data-analysis. Chi-square test was applied for determining frequencies of anatomical variants also, Pearson-correlation was used to correlate the symptoms of sinusitis with anatomical variants. The p-value of ≤ 0.05 was considered as significant.

## RESULTS

**Fig.1:**
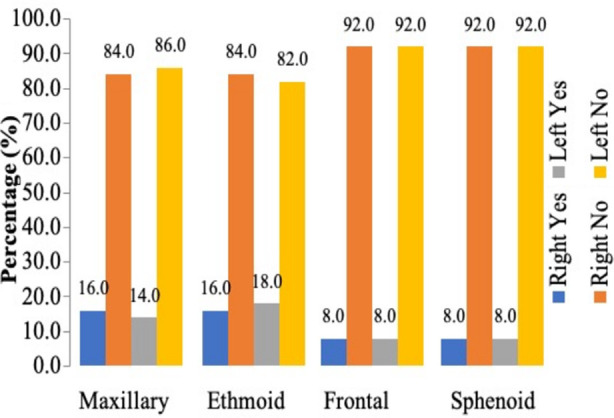
Distribution of involved sinuses.

The total number of subjects was 50, out of which 34 were males and 16 were females with the mean age of 42.68±18.22 years. Overall, most common sinonasal anatomical variants were found to be as agger-nasi cells (64%), DNS (56%), CB (46%), haller cells (10%) and onodi cells (10%). The distribution of involved sinuses is shown in Fig.1.

Correlation between anatomic variants of sinonasal region with symptoms of sinusitis is shown in Table-I. Correlation between anatomic variants with involved sinus and nasal obstruction (Table-II) in which it was observed that significant positive correlation existed between right-sided nasal obstruction with sphenoid-sinusitis (r=0.309, p=0.029). Significant negative correlation existed between right-sided nasal obstruction with agger-nasi (r=-0.305, p=0.031) as well as between left-sided nasal obstruction with agger-nasi cells (r=-0.336, p=0.017).

**Table-I:** Correlation between anatomic variants of sinonasal region with symptoms of sinusitis.

Variable		Nasal Obstruction		Facial-pain		Rhinorrhea	

		ρ	p-value	ρ	p-value	ρ	p-value
Nasal Septum	Septal Deviation	-0.075	0.603	-0.184	0.200	-0.242	0.091
	Septal Bony Spur	0.062	0.667	-0.079	0.586	-0.124	0.932
Turbinates	Superior CB	0.229	0.110	-0.042	0.771	-0.048	0.743
	Middle CB	0.229	0.110	-0.124	0.390	-0.174	0.227
	Paradoxical Middle Concha	-0.089	0.538	-0.042	0.771	-0.048	0.743
Uncinate Process	Deviation of Upper Edge	-0.127	0.378	-0.060	0.678	-0.068	0.639
	Pneumatization	-0.089	0.538	-0.042	0.771	-0.048	0.743
Ethmoid Air-Cells	Agger-Nasi	-0.275	0.54	-0.240	0.094	-0.167	0.247
	Haller	-0.059	0.682	-0.098	0.497	-0.111	0.442
	Onodi	-0.208	0.147	-0.098	0.497	-0.111	0.442
Other Variants	Maxillary-Septa	-0.089	0.538	-0.042	0.771	-0.048	0.743
	Hypoplastic Frontal Sinus	-0.332	0.018	-0.202	0.159	-0.229	0.110
	Asymmetry of Both Cavities of Sphenoid Sinus	-0.151	0.296	0.072	0.617	-0.145	0.313

p-value significant at ≤ 0.05; ρ=rho=Pearson correlation co-efficient.

**Table-II:** Correlation between anatomic variants with involved sinus and nasal obstruction.

Variable	Nasal Obstruction			

	Right		Left	

	ρ	p-value	ρ	p-value
Maxillary-sinusitis	0.214	0.136	0.262	0.066
Ethmoid-sinusitis	0.092	0.524	0.172	0.233
Frontal-sinusitis	0.144	0.317	0.309 *	0.029
Sphenoid-sinusitis	0.309 *	0.029	0.309 *	0.029
Agger-Nasi	-0.305 *	0.031	-0.336 *	0.017
Onodi	-0.208	0.147	-0.208	0.147
CB	0.050	0.732	0.127	0.378
Haller	-0.020	0.892	-0.059	0.682
DNS convexity towards	0.134	0.355	-0.117	0.420
DNS with Spur	0.172	0.233	-0.009	0.953

* Statistically significant at ≤ 0.05; ρ=rho=Pearson correlation co-efficient

Correlation between anatomic variants with involved sinus and facial pain (Table-III) in which significant positive correlation existed between right-sided facial pain with maxillary-sinusitis (r=0.475, p=<0.001), sphenoid-sinusitis (r=0.457, p=0.001). Significant positive correlation existed between left-sided facial pain with maxillary-sinusitis (r=0.306, p=0.031), frontal-sinusitis (r=0.457, p=0.001), sphenoid-sinusitis (r=0.457, p=0.001).

**Table-III:** Correlation between anatomic variants with involved sinus and facial pain.

Variable	Facial-Pain			

	Right		Left	

	ρ	p-value	ρ	p-value
Maxillary-sinusitis	0.475 *	<0.001	0.306 *	0.031
Ethmoid-sinusitis	0.072	0.617	0.246	0.086
Frontal-sinusitis	0.185	0.199	0.457 *	0.001
Sphenoid-sinusitis	0.457 *	0.001	0.457 *	0.001
Agger-Nasi	-0.255	0.074	-0.272	0.056
Onodi	-0.098	0.497	-0.098	0.497
CB	-0.044	0.760	-0.090	0.533
Haller	-0.087	0.548	-0.098	0.497
DNS convexity towards	0.037	0.799	-0.193	0.179
DNS with Spur	0.054	0.711	-0.157	0.277

* Statistically significant at ≤ 0.05; ρ=rho=Pearson correlation co-efficient

Correlation between anatomic variants with involved sinus and rhinorrhea is summarized in Table-IV in which significant positive correlation existed between right-sided rhinorrhea with maxillary-sinusitis (r=0.582, p=<0.001), frontal-sinusitis (r=0.393, p=0.005), sphenoid-sinusitis (r=0.393, p=0.005). Significant positive correlation existed between left-sided rhinorrhea with maxillary-sinusitis (r=0.442, p=0.001), ethmoid-sinusitis (r=0.364, p=0.009), frontal-sinusitis (r=0.393, p=0.005), sphenoid-sinusitis (r=0.639, p=<0.001).

**Table-IV:** Correlation between anatomic variants with involved sinus and rhinorrhea.

Variable	Rhinorrhea			

	Right		Left	

	ρ	p-value	ρ	p-value
Maxillary-sinusitis	0.582 *	<0.001	0.442 *	0.001
Ethmoid-sinusitis	0.218	0.128	0.364 *	0.009
Frontal-sinusitis	0.393 *	0.005	0.393 *	0.005
Sphenoid-sinusitis	0.393 *	0.005	0.639 *	<0.001
Agger-Nasi	-0.183	0.203	-0.200	0.164
Onodi	-0.111	0.442	-0.111	0.442
CB	-0.229	0.110	-0.136	0.346
Haller	-0.098	0.497	-0.111	0.442
DNS convexity towards	0.0	>0.999	-0.218	0.128
DNS with Spur	0.017	0.905	-0.177	0.219

* Statistically significant at ≤ 0.05; ρ=rho=Pearson correlation co-efficient

## DISCUSSION

The extensive surgical techniques and prolonged hospital visits have been replaced by a minimally invasive procedure called FESS. Although, literature has described outstanding results with FESS. However, presence of anatomical variants and the close proximity of sinonasal region to vital structures makes this region highly vulnerable to surgical trauma. Researchers believe that anatomical variants are causative factor for sinus mucosal disease but at the same time the results in this regard are controversial. A study revealed frequency of DNS (88.2%), CB (76.4), agger-nasi cells (7%), haller cells (3.5%), onodi cells (1.6%) and stated that there was no correlation between anatomic variants and sinusitis.^9^ Moreover, Adeel et al., studied various sinonasal anatomic variants as DNS (26%), CB (18.2%), haller cells (9.1%), onodi cells (7.8%) and emphasized on their surgical significance.^3^ In addition, Usman et al., observed frequency of variants as DNS (31%), CB (18.9%), UP variations (12%), agger-nasi cells (6.8%), septal spur (4%) and haller cells (3.7%) and have stressed on pre-operative evaluation of variants to minimize surgical complications.^10^

One of the studies^11^ has stated that there was significant association existed between uncinate bulla and ethmoid bulla with sinus-mucosal infection also, reported frequency of variants as DNS (59.5%), CB (67.5%) and agger-nasi cells (74.8%). Another study^12^ reported that strong correlation was recognized between agger-nasi cells, DNS, turbinate variants with rhinosinusitis also, stated incidence of DNS (20.0%), CB (11.7%) and agger-nasi cells (78.3%). These results were compatible with the results of Madani et al., and Suri et al., that showed there was strong association between anatomical variants and rhinosinusitis. Also, there is an impact of variants on sinus infections and hence CT is the best tool for evaluation of sinonasal region.^13^,^14^

The present study yielded statistically significant correlation existed between bilateral maxillary, frontal and ethmoid sinusitis with rhinorrhea. However, no correlation was found between anatomical variants and symptoms of sinusitis except significant negative correlation existed between bilateral agger nasi cells and nasal obstruction with incidence of agger-nasi cells (64%), DNS (56%), CB (46%), haller cells (10%), onodi cells (10%). The overall results revealed insignificant correlation between anatomical variations and sinus mucosal disease except for bilateral agger-nasi cells.

Anatomy of nasal cavity and PNS display considerable variations. These variations depend on various factors like age, race, gender, geography, and ethnicity, only few of these anatomical variations have been associated to pathogenesis of inflammatory sinus-mucosal diseases. Comprehensive understanding of sinonasal anatomic variants is not only relevant diagnostically, but also play an important role in reducing the rate of intraoperative also post-operative complications of FESS/skull-base surgery. CT-PNS is playing important role in evaluating the extent and type of anatomical variations in sinus region hence, it provides road map for ENT surgeons.^9^,^15^-^19^

### Limitation of the study

This is a single-centered study, thus for more better and generalized results larger population size and multicenter options must be taken to authenticate study further.

## CONCLUSION

In nasal cavity and PNS there are a multitude of anatomical variants. The variable anatomy of sinonasal region can disturb sinus drainage pathway leading to infection. Out of those anatomical alterations some variants are so common that these variations are mostly observed in majority of population around the globe. The results of present study showed significant correlation existed between bilateral maxillary, frontal, ethmoid sinusitis and rhinorrhea. However, no correlation was found between anatomical variants and symptoms of sinusitis except significant negative correlation existed between bilateral agger nasi cells and nasal obstruction. It was concluded that there is no significant difference in the occurrence of any of sinonasal anatomic variants among patients with sinus-mucosal disease. Therefore, analysis of every routine CT-PNS attained for rhino-sinusitis or for the presence of diverse anatomic variants is of questionable value unless surgical option is planned. It is significant to be conscious of certain anatomic variants, such as ethmoidal cells including onodi cells, supraorbital cells, haller cells, and many other for those patients who are planning to undergo FESS/skull-base surgery. Failure to identify these anatomic variants is related with higher rate of operative complications.

### Author`s Contribution:

**MFQ** conceived, designed and prepared the manuscript and she is also the responsible and accountable for the accuracy study.

**AU** critical analysis, review and final approval of manuscript.
